# Oxidase-Like Catalytic Performance of Nano-MnO_2_ and Its Potential Application for Metal Ions Detection in Water

**DOI:** 10.1155/2019/5416963

**Published:** 2019-11-03

**Authors:** Kai Sun, Qingzhu Liu, Rui Zhu, Qi Liu, Shunyao Li, Youbin Si, Qingguo Huang

**Affiliations:** ^1^Anhui Province Key Laboratory of Farmland Ecological Conservation and Pollution Prevention, School of Resources and Environment, Anhui Agricultural University, 130 Changjiang West Road, Hefei, 230036 Anhui, China; ^2^College of Resources and Environmental Sciences, Nanjing Agricultural University, Nanjing 210095, China; ^3^Department of Crop and Soil Sciences, University of Georgia, Griffin, GA 30223, USA

## Abstract

Certain nano-scale metal oxides exhibiting the intrinsic enzyme-like reactivity had been used for environment monitoring. Herein, we evaluated the oxidase-mimicking activity of environmentally relevant nano-MnO_2_ and its sensitivity to the presence of metal ions, and particularly, the use of MnO_2_ nanozyme to potentially detect Cu^2+^, Zn^2+^, Mn^2+^, and Fe^2+^ in water. The results indicated the oxidase-like activity of nano-MnO_2_ at acidic pH-driven oxidation of 2,6-dimethoxyphenol (2,6-DMP) via a single-electron transfer process, leading to the formation of a yellow product. Notably, the presence of Cu^2+^ and Mn^2+^ heightened the oxidase-mimicking activity of nano-MnO_2_ at 25°C and pH 3.8, showing that Cu^2+^ and Mn^2+^ could modify the reactive sites of nano-MnO_2_ surface to ameliorate its catalytic activity, while the activity of MnO_2_ nanozyme in systems with Zn^2+^ and Fe^2+^ was impeded probably because of the strong affinity of Zn^2+^ and Fe^2+^ toward nano-MnO_2_ surface. Based on these effects, we designed a procedure to use MnO_2_ nanozyme to, respectively, detect Cu^2+^, Zn^2+^, Mn^2+^, and Fe^2+^ in the real water samples. MnO_2_ nanozyme-based detecting systems achieved high accuracy (relative errors: 2.2–26.1%) and recovery (93.0–124.0%) for detection of the four metal ions, respectively. Such cost-effective detecting systems may provide a potential application for quantitative determination of metal ions in real water environmental samples.

## 1. Introduction

In recent years, considerable attention has been paid to the applications of artificial nanomaterials as nanozymes in mimicking the intrinsic catalytic function of natural enzymes due to their unique structural, electrical, and optical properties, as well as remarkable catalytic activities [1–3]. Compared with the natural enzymes, the artificial nanozymes exhibited higher robustness and stability under harsh conditions, lower production cost, simpler storage conditions, and more effective catalytic activity [4–6]. At present, nanozymes are primarily composed of artificial metal and metal oxide nanomaterials that can mimic the catalytic activities of natural peroxidases and/or oxidases [3, 7]. For instance, the intrinsic peroxidase-/oxidase-like activities of Au-Ag, CeO_2_, MnFe_2_O_4_, NiO, and V_2_O_5_ nanoparticles have been used in various applications ranging from biosensing and immunoassay to environment monitoring [3, 7–10]. Liu et al. reported the oxidase-mimicking activity of CeO_2_ nanoparticles by fluoride capping, such nanozymes could detect micromolar levels of F^−^ in water and toothpastes [11].

It is well documented that MnO_2_ nanomaterial had the intrinsic enzyme-like activity to catalyze the chromogenic reaction of substrates, which could be used as a nanozyme indicator for bioimaging, biosensing, and delivery of single-stranded DNA and drugs [3, 12, 13]. In particular, chromogenic reactions by nano-MnO_2_ have been developed using dissolved O_2_ as the oxidant, avoiding the use of H_2_O_2_ [14], thus providing easy and rapid detecting systems for quantitative analysis of any substances that can serve either as the accelerator or inhibitor of the chromogenic reactions [15, 16]. Such systems could be used for real environmental water samples, but their potential application for quantitative determination of metal ions has been rarely explored.

The contamination of metal ions has always been a focus of concern [17]. Toxic metal ions (such as Cu^2+^, Zn^2+^, Pb^2+^, and Fe^2+^) having toxicity level greater than safety levels can cause acute toxicities to most aquatic biota [18]. Thus, having ways that can easily and rapidly detect these metal ions in water matrices is vital to protect wild species and human health. A instrumental method can be used to directly detect these metal ions in water samples, such as inductively coupled plasma mass spectrometry (ICPMS), but such methods are usually expensive and time-consuming and require expertise to operate [19, 20].

Recently, the enzyme-like activity of environmentally relevant nano-MnO_2_ has proven to be highly effective for sensing applications [16, 21, 22]. In this study, nano-MnO_2_ was chosen as the natural oxidase mimic owing to its outstanding redox chemistry, stability, and biocompatibility properties [23–25]. We systematically evaluated the oxidase-like activity of nano-MnO_2_ in catalyzing the chromogenic reaction of 2,6-dimethoxyphenol (2,6-DMP), and identified the influence of Cu^2+^, Zn^2+^, Mn^2+^, and Fe^2+^, based on which methods were developed to, respectively, detect these metal ions in environmental water samples using MnO_2_ nanozyme-2,6-DMP detecting systems.

## 2. Materials and Methods

### 2.1. Chemicals and Materials

**Figure 1:**
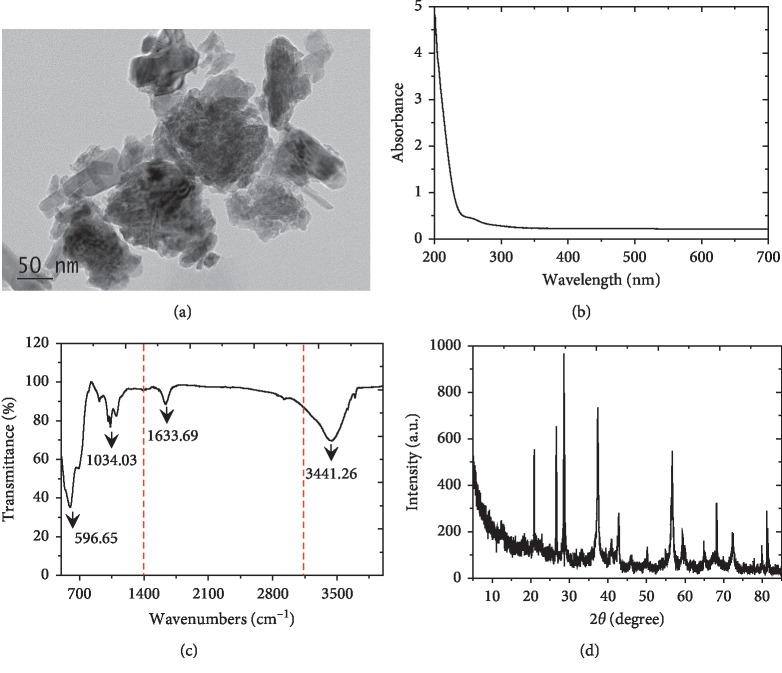
MnO_2_ nanomaterial was characterized by (a) TEM imaging, (b) UV-Vis spectra, (c) FTIR spectra, and (d) XRD pattern to determine the size, morphology, group, and crystalline structure of nano-MnO_2_.

Nano-scale MnO_2_ (≥99.9%) was obtained from DK Nano Technology Co., Ltd. (Beijing, China). The characteristics of nano-MnO_2_ are shown in Figure 1. The size and morphology of the nano-MnO_2_ were analyzed using a transmission electron microscope (TEM, JEM-200CX). The spectral characteristics of nano-MnO_2_ were investigated using a UV-Vis spectroscope (Shimadzu, UV-2550) and a Fourier-transform infrared spectroscope (Thermo Scientific, NICOLET iS50 FTIR). The phase of the nano-MnO_2_ was measured over the 2*θ* range from 5 to 85 degrees using an X-ray diffractometer (XRD, Thermo X'TRA).

2,6-DMP (CAS: 91-10-1) was purchased from Energy Chemical Technology Co., Ltd (Shanghai, China). Metal sulfates (*i.e.*, MgSO_4_, CuSO_4_, Al_2_(SO_4_)_3_, ZnSO_4_, MnSO_4_·H_2_O, FeSO_4_·7H_2_O, and PbSO_4_) were obtained from Shanghai Aladdin Bio-Chem Technology Co., Ltd. Stock solutions of metal ions (100 mmol·L^−1^) were prepared in Milli-Q ultrapure water (18.2 MΩ·cm) and stored at 4°C. We had previously evaluated the effects of K^+^, Na^+^, Ag^+^, Co^2+^, Hg^2+^, Ca^2+^, Cd^2+^, and Fe^3+^ on the oxidase-like activity of nano-MnO_2_. The buffer used in this study was a citrate-phosphate buffer solution (C-PBS: 10 mmol·L^−1^ citric acid and 10 mmol·L^−1^ Na_2_HPO_4_, pH 3.8) adjusted with HCl and NaOH. All the other chemicals were of analytical reagent grade and used as received.

### 2.2. Assessment of the Enzyme-Like Activity of Nano-MnO_2_

To assess the enzyme-like activity of nano-MnO_2_, MnO_2_ nanoparticles were tested in 10 mL of a C-PBS (10 mmol·L^−1^, pH 3.8) buffer at room temperature (25°C) containing 1.0 mmol·L^−1^ 2,6-DMP as the chromogenic substrate and naturally dissolved O_2_ as the cofactor [14, 26]. After the nano-MnO_2_ (0.1 mg·mL^−1^) had been mixed thoroughly with the 2,6-DMP reaction solution, the absorbance was immediately measured at 468 nm using a UV-Vis spectrophotometer (Shanghai Lengguang 722S) in a quartz cuvette with a 1 cm light path. The solution was monitored every 20 s for 3 min by recording the change of absorbance value at 468 nm. One unit of nano-MnO_2_ activity (U·mL^−1^) is defined as the amount of nanozyme that causes one unit of absorbance change per minute at 468 nm in C-PBS (10 mmol·L^−1^, pH 3.8) buffer containing 1.0 mmol·L^−1^ 2,6-DMP. Therefore, the oxidase-mimicking activity of nano-MnO_2_ can be calculated through the rate of absorbance change. The same solution free of nano-MnO_2_ was used as the blank control. All experiments were performed in triplicate.

### 2.3. Effect of Different Factors on the Enzyme-Like Activity of Nano-MnO_2_

To evaluate the influence of nano-MnO_2_ dosage on 2,6-DMP oxidation, the reaction was conducted in a 50 mL flask containing 1.0 mmol·L^−1^ 2,6-DMP and nano-MnO_2_ varying between 0.005 and 0.32 mg·mL^−1^ in 10 mL C-PBS (10 mmol·L^−1^) at 25°C and pH 3.8. The effect of the substrate concentration on the chromogenic reaction was also performed in a 50 mL flask containing 0.1 mg·mL^−1^ nano-MnO_2_ and 2,6-DMP at a concentration varying between 0.005 and 1.0 mmol·L^−1^ in 10 mL C-PBS. The reaction kinetics parameters *K*_m_ and *v*_max_ were calculated by the Lineweaver–Burk plot of the Michaelis–Menten kinetics equation:

(1) $$\begin{array}{c} \frac{1}{v}=\left(\;\frac{K_{\text{m}}+\left[S\right]}{v_{\max}\cdot \left[S\right]}\right), \end{array}$$

where *v* is the reaction velocity, [*S*] is the substrate concentration, *K*_m_ is the Michaelis constant, and *v*_max_ is the maximal reaction velocity.

Experimental procedures similar to those described above were used to explore the effects of pH and temperature on the enzyme-like activity of nano-MnO_2_. The reactions were carried at different pH and a wide range of temperature. For studying the pH effect, 10 mL of 10 mmol·L^−1^ C-PBS (pH 2.0–10.0) buffer containing 1.0 mmol·L^−1^ 2,6-DMP was mixed with 0.1 mg·mL^−1^ nano-MnO_2_ at room temperature (25°C). For studying the effect of temperature, 10 mL of 10 mmol·L^−1^ C-PBS (pH 3.8) buffer containing 1.0 mmol·L^−1^ 2,6-DMP was mixed with 0.1 mg·mL^−1^ nano-MnO_2_ at a temperature ranging from 10°C to 90°C. Absorbance was recorded at 468 nm at 20 s intervals. All experiments were performed in triplicate.

### 2.4. Enzyme-Like Activity of Nano-MnO_2_ for Detecting Metal Ions in Water

A series of mixtures containing different metal ions (*i.e*., Mg^2+^, Cu^2+^, Al^3+^, Zn^2+^, Mn^2+^, Fe^2+^, and Pb^2+^) and nano-MnO_2_ (0.1 mg·mL^−1^) in 10 mL of C-PBS buffer (pH 3.8) were equilibrated at room temperature (25°C), and then C-PBS containing 1.0 mmol·L^−1^ 2,6-DMP was added to each of the mixtures and then monitored using a UV-Vis spectrophotometer at 468 nm. All experiments were performed in triplicate.

Based on the above results, MnO_2_ nanozyme-2,6-DMP reaction systems for, respectively, detecting Cu^2+^, Zn^2+^, Mn^2+^, and Fe^2+^ in real environmental water matrices were also assessed. 10-fold dilution of real samples (pond water 1 and 2 from Anhui Agricultural University campus) in C-PBS buffer (10 mmol·L^−1^, pH 3.8) were spiked with Cu^2+^, Zn^2+^, Mn^2+^, or Fe^2+^ (0.002, 0.01, 0.05, and 0.25 mmol·L^−1^) and were then detected using the described MnO_2_ nanozyme-2,6-DMP-sensing systems following the same process described above. Additionally, these samples were also determined by ICPMS (6300 Series, Thermo Fourier, USA) for comparison. All experiments were performed in quintuplicate.

### 2.5. Statistical Analysis

All data were processed with Excel 2010 (Microsoft, Redmond, WA). Each data point in the figures and tables represents an average value. The standard deviation of replicate samples is shown in the figures as an error bar.

## 3. Results and Discussions

### 3.1. Oxidase-Like Activity of Nano-MnO_2_

**Figure 2:**
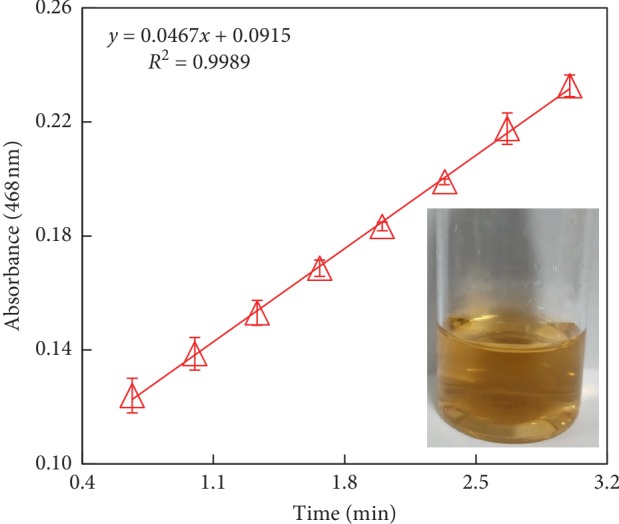
Oxidase-like activity of nano-MnO_2_ for catalyzing the chromogenic reaction of 2,6-DMP in C-PBS buffer at 25°C and pH 3.8. Reaction conditions: Nano-MnO_2_ = 0.1 mg·mL^−1^; 2,6-DMP = 1.0 mmol·L^−1^. Error bars represent the standard deviation (*n* = 3).

To assess the intrinsic enzyme-mimicking activity of nano-MnO_2_, 2,6-DMP was chosen as the chromogenic substrate in the standard oxidation reaction, and the reaction kinetics was tested at 468 nm corresponding to the oxidized 2,6-DMP. The change of absorbance over time by the oxidation of 2,6-DMP in C-PBS buffer at 25°C and pH 3.8 is shown in Figure 2. Nano-MnO_2_ could catalyze the colorless 2,6-DMP to form a chromogenic product (a yellow product, *i.e.*, 3,3′,5,5′-tetramethyl-4,4′-diphenoquinone) with a change in absorbance via the radical-based C-C self-coupling mechanism, like laccase-mediated oxidative coupling reactions of 2,6-DMP under the same conditions [26, 27]. The absorbance changes linearly with time under the tested conditions (*R*^2^ > 0.99), and the oxidase-like activity of nano-MnO_2_ was calculated to be 0.047 U·mL^−1^. The oxidative coupling of 2,6-DMP catalyzed by nano-MnO_2_ was described as follows: first, 2,6-DMP was adsorbed onto the reactive sites of nano-MnO_2_ surface, followed by the single-electron oxidation of 2,6-DMP by nano-MnO_2_, leading to the formation of chromogenic product and the release of Mn^2+^ from the nanoparticle surface [14, 28].

The role of dissolved O_2_ in the oxidation of 2,6-DMP was evaluated by purging the reaction solution with N_2_, resulting in a decrease on the oxidase-like activity of nano-MnO_2_. This revealed that dissolved O_2_ acted as an electron acceptor in the catalytic reactions [14, 29]. This result is in agreement with an earlier report that indicated the oxidation of a substrate in the absence of H_2_O_2_ via bovine serum albumin- (BSA-) stabilized MnO_2_ nanoparticles [30]. Additionally, the stability of nano-MnO_2_ in the reaction system was also studied over a one-month storage period. With the increase in storage time, the release of Mn^2+^ increased mildly, but no significant difference in the oxidation of 2,6-DMP was detected, implying that the capacity of nano-MnO_2_ to oxidize 2,6-DMP exhibits a high stability. These results demonstrated that nano-MnO_2_ possessed a stable oxidase-like activity to catalyze the chromogenic reaction of 2,6-DMP at 25°C and pH 3.8 in the absence of H_2_O_2_.

### 3.2. Effects of Nano-MnO_2_ and Substrate Concentration on 2,6-DMP Oxidation

**Figure 3:**
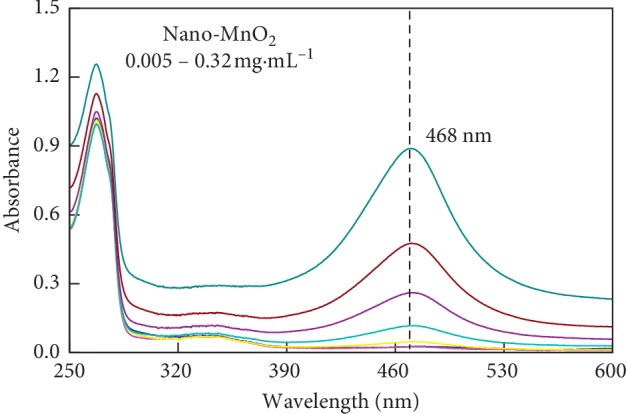
UV-Vis absorption spectra of 2,6-DMP reacted with different nano-MnO_2_ concentrations at the wavelength of 250–600 nm. Reaction conditions: Nano-MnO_2_ = 0.005–0.32 mg·mL^−1^; 2,6-DMP = 1.0 mmol·L^−1^.

**Figure 4:**
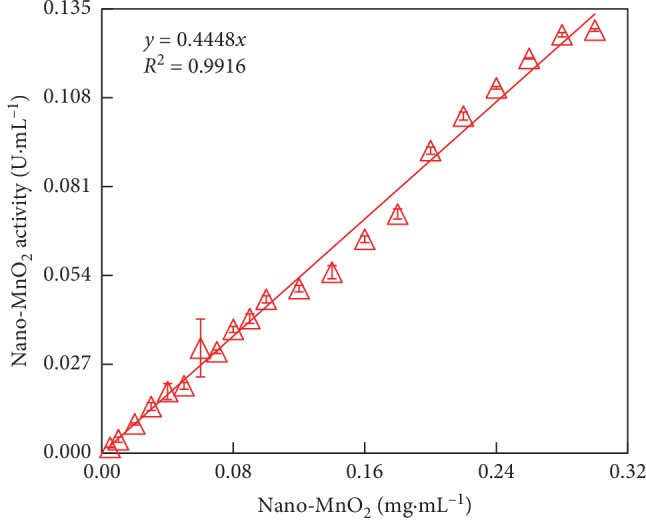
Concentration-dependent kinetic study of the oxidase-like activity of nano-MnO_2_ for a 3 min reaction. Reaction conditions: [Nano-MnO_2_] = 0.005–0.3 mg·mL^−1^, [2,6-DMP] = 1.0 mmol·L^−1^. Error bars represent the standard deviation (*n* = 3).

We further assessed the influence of nano-MnO_2_ concentration on 2,6-DMP oxidation catalyzed by MnO_2_ nanozyme by UV-Vis spectrophotometry. As shown in Figure 3, the oxidation of 2,6-DMP catalyzed by MnO_2_ nanozyme showed a distinct absorbance peak at the wavelength of 468 nm, and the increase of this absorbance over time was obvious resulting from 2,6-DMP oxidation (Figure 2). The variation of the absorbance peak was observed by adding different concentrations of MnO_2_ nanozyme (Figure 3). Increasing the concentration of nano-MnO_2_ from 0.005 to 0.3 mg·mL^−1^ resulted in a liner increase in the oxidase-like activity of nano-MnO_2_ (0.002–0.126 U·mL^−1^) in oxidizing 2,6-DMP (Figure 4). According to the correlation of the nano-MnO_2_ concentration and its oxidase-like activity, the apparent pseudo-second-order rate constant was determined to be 0.445 U·mg^−1^ (*R*^2^ = 0.992). These results demonstrated that increasing the concentration of nano-MnO_2_ facilitated the oxidase-like activity of nano-MnO_2_ to catalyze the oxidation of 2,6-DMP.

**Figure 5:**
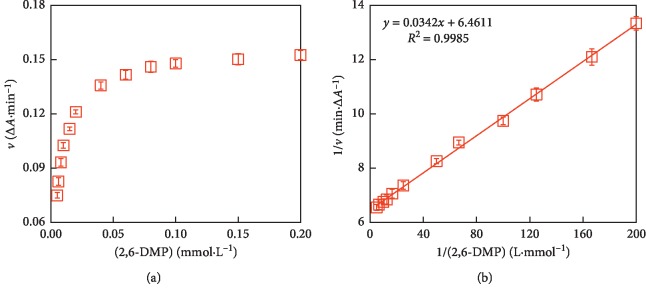
Studies of kinetic reaction parameters *K*_m_ and *v*_max_. (a) The Michaelis–Menten curve for the oxidase-like activity of nano-MnO_2_. (b) Lineweaver–Burk plot of 2,6-DMP oxidation was made from the Michaelis–Menten curve. Reaction conditions: Nao-MnO_2_ = 0.1 mg·mL^−1^; 2,6-DMP = 0.005–0.2 mmol·L^−1^. Error bars represent the standard deviation (*n* = 3).

For discussing the catalytic mechanism and obtaining the steady-state kinetic parameters, the initial reaction rate (1 min) of 2,6-DMP oxidation catalyzed by nano-MnO_2_ was investigated with the initial 2,6-DMP concentration varying between 0.005 and 0.2 mmol·L^−1^. A hyperbolic relationship between the substrate concentration and the rate of reaction (*v*) was revealed in Figure 5(a), like the typical Michaelis–Menten curve. The apparent enzyme kinetic parameters such as *K*_m_ and *v*_max_ values could be calculated by Lineweaver–Burk plot (Figure 5(b)). From the kinetic analysis, it was found that MnO_2_ nanozyme showed a high affinity towards 2,6-DMP. The *K*_m_ and *v*_max_ values were 0.005 and 0.155 (*R*^2^ = 0.999), respectively. Combining with previous studies on artificial metal oxide-based nanozymes [24, 31, 32], MnO_2_ nanoparticles are promising nanomimetics for oxidase. It is noted that the oxidase-like activity of nano-MnO_2_ and the steady-state kinetic parameter values were investigated at an acidic pH (pH 3.8) because of its limited oxidase-like activity at physiological or basic pH.

### 3.3. Effects of pH and Temperature on 2,6-DMP Oxidation

**Figure 6:**
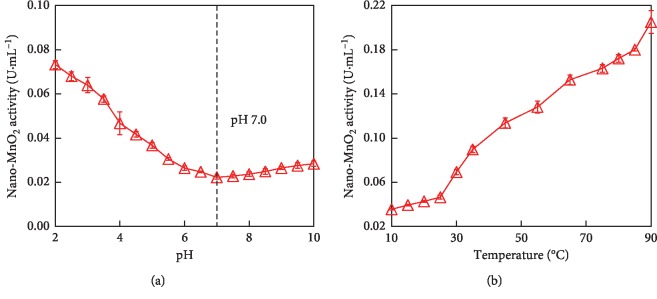
pH and temperature dependent the oxidase-like activity of nano-MnO_2_ for catalyzing the oxidation of 2,6-DMP. (a) Reaction conditions: Nano-MnO_2_ = 0.1 mg·mL^−1^; 2,6-DMP = 1.0 mmol·L^−1^, pH 2.0–10.0. (b) Reaction conditions: Nano-MnO_2_ = 0.1 mg·mL^−1^; 2,6-DMP = 1.0 mmol·L^−1^; Temperature = 10–90°C. Error bars represent the standard deviation (*n* = 3).

Similar to the natural oxidase, the catalytic activity of MnO_2_ nanozyme is also dependent on pH and temperature. As shown in Figure 6, the catalytic activity of MnO_2_ nanozyme decreased with the rise of reaction pH from 2.0 to 7.0, whereas the oxidase-like activity of MnO_2_ nanozyme increased with the rise of reaction temperature from 10°C to 90°C. It was found that only 0.022 U·mL^−1^ of nano-MnO_2_ activity was retained at pH 7.0, while 0.205 U·mL^−1^ of activity was retained even at 90°C. As the reaction pH increasing from 7.0 to 10.0, the oxidase-like activity of nano-MnO_2_ had not exhibited an obvious variation. As the temperature increased from 10°C to 25°C, the catalytic activity of nano-MnO_2_ was mildly enhanced. It was noted that as the temperature increased from 30°C to 90°C, the catalytic activity rapidly increased. Temperature varying in the range of 10–25°C had little impact on the final colorimetric signal. Change in pH and temperature had not resulted in inactivation of MnO_2_ nanozyme. These results indicated that the oxidase-like activity of nano-MnO_2_ exhibited a wide range of pH and thermal stability, unlike the natural oxidase [33, 34].

### 3.4. Metal Ions Induced the Effect of MnO_2_ Nanozyme Activity

**Figure 7:**
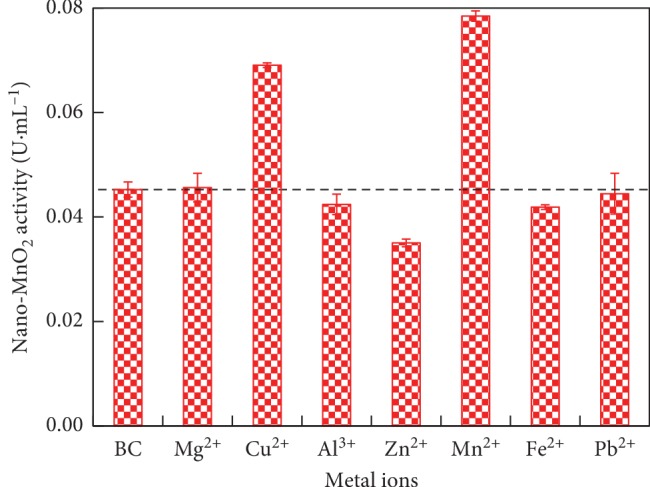
Role of metal ions (*i.e.*, Mg^2+^, Cu^2+^, Al^3+^, Zn^2+^, Mn^2+^, Fe^2+^, and Pb^2+^) on the oxidase-like activity of nano-MnO_2_ for catalyzing the oxidation of 2,6-DMP. Reaction conditions: Nano-MnO_2_ = 0.1 mg·mL^−1^; 2,6-DMP = 1.0 mmol·L^−1^; Metal ion = 0.01 mmol·L^−1^. Error bars represent the standard deviation (*n* = 3).

Simply and accurately detecting metal ions is of great significance in the aqueous environment. Several nanozymes had been used to detect metal ions (*i.e.*, Hg^2+^ and Pb^2+^) due to their intrinsic advantages and high stability under harsh conditions [35–37]. In this study, the selectivity of MnO_2_ nanozyme activity was evaluated in the presence of various metal ions including Mg^2+^, Cu^2+^, Al^3+^, Zn^2+^, Mn^2+^, Fe^2+^, and Pb^2+^ in 10 mL C-PBS buffer at 25°C and pH 3.8. As shown in Figure 7, the oxidase-like activity of nano-MnO_2_ was 0.045 U·mL^−1^ in the blank control (BC, *i.e.*, metal ion-free) samples. Compared with BC, the activity of MnO_2_ nanozyme was significantly enhanced in the presence of Cu^2+^ and Mn^2+^ (*P* < 0.01), whereas the presence of Zn^2+^ and Fe^2+^ obviously suppressed the activity of MnO_2_ nanozyme (*P* < 0.05). Interestingly, there was no significant interference on the activity of MnO_2_ nanozyme in aqueous solution by other metal ions. These results implied that MnO_2_ nanozyme might be used to, respectively, detect Cu^2+^, Mn^2+^, Zn^2+^, and Fe^2+^ in aquatic environment. However, the selectivity of MnO_2_ nanozyme toward Cu^2+^, Mn^2+^, Zn^2+^, and Fe^2+^ against other ions needs further studies due to the complexity of valence states of metal elements in the nanoparticles.

Previous studies had also indicated that certain metal ions could effectively upregulate/downregulate the activity of nanozymes through surface deposition and metallophilic interactions [38–40]. For MnO_2_ nanozyme detecting systems, the substrate (2,6-DMP) was transformed into a chromogenic product, serving as a signal amplifier. The presence of Cu^2+^ and Mn^2+^ enhanced the activity of MnO_2_ nanozyme, likely because these metal ions modified the reactive sites of nano-MnO_2_ surface [41, 42]. First, Cu^2+^ and/or Mn^2+^ ions reacted with citrate to form metal ion-citrate complex, subsequently the complex dispersed onto the surface of nano-MnO_2_, and thus changed the surface properties of nano-MnO_2_, thereby enhancing its oxidase-like activity [43, 44]. On the contrary, the suppressive activity on MnO_2_ nanozyme in the presence of Zn^2+^ and Fe^2+^ occurred probably owing to the strong affinity of Zn^2+^ and Fe^2+^ toward the nano-MnO_2_ surface via the electrostatic attractions or metal ion-multivalent Mn interactions [36, 39, 40]. The binding affinity of MnO_2_ nanozyme for Zn^2+^ and Fe^2+^ was very high. The adsorption of Zn^2+^ and Fe^2+^ onto the MnO_2_ nanozyme impeded the electron transfer to 2,6-DMP, thus diminishing the oxidase-like activity of nano-MnO_2_ [39]. Additionally, the control samples free of MnO_2_ nanozyme with the metal ion present did not show the oxidase-like activity towards O_2_-2,6-DMP during the incubation period.

### 3.5. MnO_2_ Nanozyme-Based Reaction Systems for Detecting Cu^2+^, Zn^2+^, Mn^2+^, or Fe^2+^

**Figure 8:**
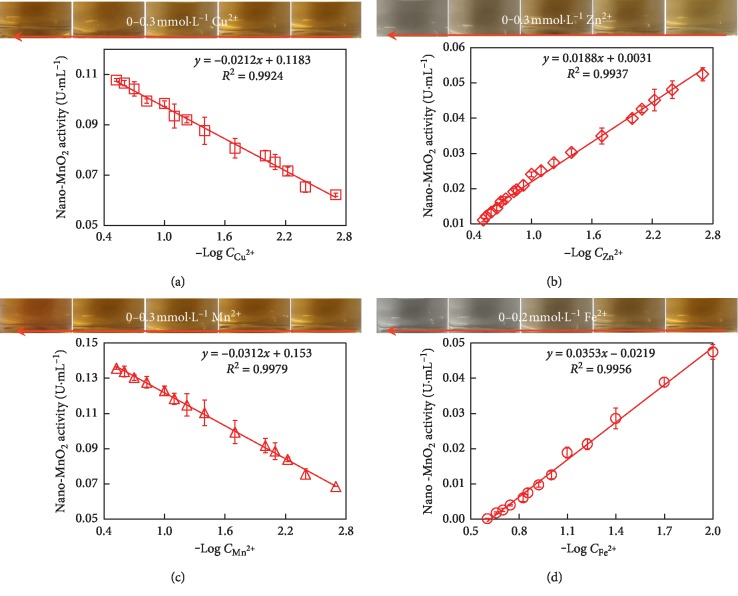
Linear correlation between the oxidase-like activity of nano-MnO_2_ and the logarithmic value of metal ion concentration (Log *C*). Reaction conditions: Nano-MnO_2_ = 0.1 mg·mL^−1^; 2,6-DMP = 1.0 mmol·L^−1^; Metal ion = 0.002–0.3 mmol·L^−1^. Error bars represent the standard deviation (*n* = 3).

As shown in Figure 8, MnO_2_ nanozyme-sensing systems were carried out by, respectively, detecting Cu^2+^, Zn^2+^, Mn^2+^, and Fe^2+^ in a concentration range of 0.002–0.3 mmol·L^−1^ by the change of MnO_2_ nanozyme activity in the presence of these metal ions. It was noted that increasing the concentrations of Cu^2+^ and Mn^2+^ resulted in a color progression from yellow to deep yellow, while increasing the concentrations of Zn^2+^ and Fe^2+^ resulted in a color progression from yellow to colorless. A linear correlation between the activities of MnO_2_ nanozyme and the logarithmic values of metal ions concentration (0.002–0.3 mmol·L^−1^) was observed (Figure 8). The activity of MnO_2_ nanozyme increased with increasing Cu^2+^ and Mn^2+^ concentrations, whereas the activity of MnO_2_ nanozyme decreased with increasing the concentrations of Zn^2+^ and Fe^2+^ ions. The slopes of the linear regression for the four metal ions (*i.e.*, Cu^2+^, Zn^2+^, Mn^2+^, and Fe^2+^) were ‒0.021, 0.019, ‒0.031, and 0.035, respectively. MnO_2_ nanozyme-2,6-DMP-sensing systems showed high sensitivity and a wide dynamic range for, respectively, detection of Cu^2+^, Zn^2+^, Mn^2+^, and Fe^2+^, allowing for a limit of detection less than 0.002 mmol·L^−1^, which was lower than the maximum levels of Cu^2+^, Zn^2+^, Mn^2+^, and Fe^2+^ (0.016, 0.015, 0.002, and 0.005 mmol·L^−1^, respectively) in drinking water permitted by the national standards GB 5749-2006 sanitary standard of China.

To further investigate the possible interaction mechanism between metal ions and MnO_2_ nanozyme, a differential UV-Vis spectrometry approach was performed [45]. The differential absorbance spectrum (DAS) could be calculated by the following equation:

(2) $$\begin{array}{c} \Delta A_{\text{DAS}}=\;A_{\text{mixture}}-\;A_{2,6-\text{DMP}}-\;A_{\text{metal ion}}, \end{array}$$

where *A*_mixture_, *A*_2,6-DMP_, and *A*_metal ion_ are, respectively, the absorbance at 250–600 nm wavelength of the mixture solution, and the corresponding reference 2,6-DMP and metal ion solution.

**Figure 9:**
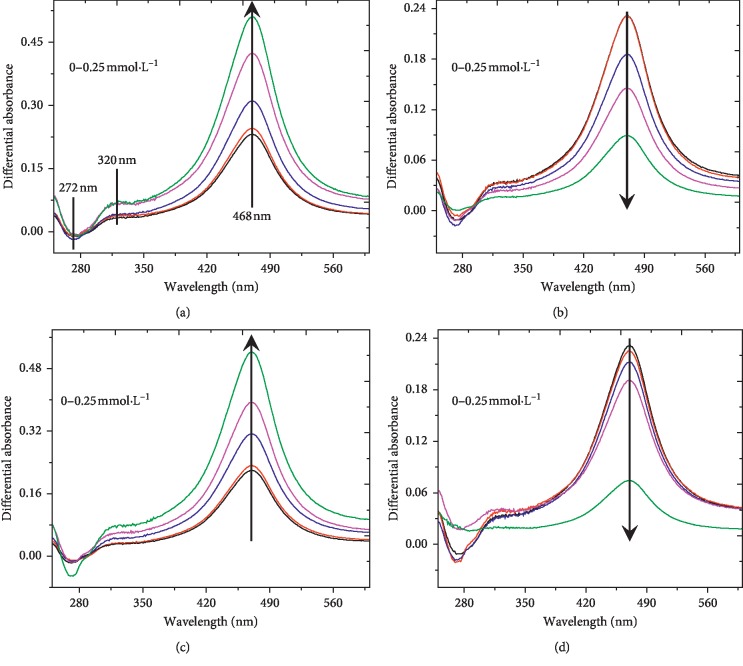
UV-Vis differential absorbance spectra (DAS) calculated on the basis of the data recorded at different metal ion concentrations (0, 0.002, 0.01, 0.05, and 0.25 mmol·L^−1^). Reaction conditions: Nano-MnO_2_ = 0.1 mg·mL^−1^; 2,6-DMP = 1.0 mmol·L^−1^; metal ion = 0–0.25 mmol·L^−1^. Error bars represent the standard deviation (*n* = 3). (a) Cu^2+^, (b) Zn^2+^, (c) Mn^2+^, and (d) Fe^2+^.

As shown in Figure 9, the DAS of four reaction systems had an intensive negative peak at 272 nm and two intensive positive peaks, respectively, at 320 and 468 nm, implying that the change of electronic density in the molecules caused by the formation of a complex and/or metal ion-multivalent Mn interactions in C-PBS buffer. On the one hand, the formation of complex between Cu^2+^/Mn^2+^ and citrate changed the surface properties of MnO_2_ nanozyme, thus facilitating its oxidase-like activity [43, 44, 46]. On the other hand, Zn^2+^ and Fe^2+^ were bound to the reactive sites of MnO_2_ nanozyme surface, leading to the hindrance of electron transfer between the MnO_2_ nanozyme and 2,6-DMP, consequently restraining the activity of MnO_2_ nanozyme [39, 40].

### 3.6. Detection of Metal Ions in Real Water Samples

In order to verify the metal sensing ability of MnO_2_ nanozyme for real environmental water samples, tests were performed with different concentrations of Cu^2+^, Zn^2+^, Mn^2+^, or Fe^2+^ spiked to pond water samples 1 and 2 from Anhui Agricultural University. First, samples were diluted 10-fold with C-PBS buffer (pH 3.8) to minimize the matrix effect. Subsequently, Cu^2+^, Zn^2+^, Mn^2+^, or Fe^2+^ at a concentration of 0.002–0.25 mmol·L^−1^ were spiked to the pond water samples. As shown in Table 1, the recoveries were 93.0–124.0% for 0.002–0.25 mmol·L^−1^ metal ions (*i.e.*, Cu^2+^, Zn^2+^, Mn^2+^, and Fe^2+^) that were spiked to the pond water 1 and 2. The concentrations of Cu^2+^, Zn^2+^, Mn^2+^, and Fe^2+^ in the pond water samples 1 and 2 were also determined by ICPMS, which did not show significant difference from that obtained by the MnO_2_ nanozyme-detecting systems. In addition, the nanozyme-sensing method exhibited stable performance at a broad range of pH and temperature, convenient for experimental applications. It is noteworthy that the response of the MnO_2_ nanozyme-sensing systems to Zn^2+^ and Fe^2+^ at high concentrations can be directly observed with the naked eye. These results confirmed that the MnO_2_ nanozyme-2,6-DMP-sensing systems may be applicable to real water environmental samples for easily and rapidly quantifying Cu^2+^, Zn^2+^, Mn^2+^, or Fe^2+^. Even so, how to improve the selectivity of MnO_2_ nanozyme for metal ions detection in water is still crucial. To achieve that, two of the following main issues need to be resolved. One is studying the catalytic performance and steady-state kinetics to uncover the interaction mechanism between MnO_2_ nanozyme and metal ions, and the other is modifying the surface of MnO_2_ nanozyme to improve its catalytic activity and environmental application in real water [3, 13, 44, 47, 48].

## 4. Conclusions

In this study, nano-MnO_2_ was used as an oxidase mimetic to catalyze the chromogenic reaction of 2,6-DMP in C-PBS buffer. The results indicated that nano-MnO_2_ possessed the oxidase-like activity with the *K*_m_ and *v*_max_ values of 0.005 and 0.155 (*R*^2^ = 0.999), respectively, at 25°C and pH 3.8. Additionally, the effect of metal ions on this colorimetric reaction catalyzed by MnO_2_ nanozyme was explored, based on which it was found that this reaction system could be used to, respectively, detect Cu^2+^, Zn^2+^, Mn^2+^, and Fe^2+^ in aqueous solution without significant interference from other factors. The detection limit for the four metal ions was less than 0.002 mmol·L^−1^ and the linear response range was 0.002–0.25 mmol·L^−1^. Use of this detecting system was demonstrated with real environmental water samples, and the results indicated that the MnO_2_ nanozyme-based sensing was simple and rapid for quantitative determination of Cu^2+^, Zn^2+^, Mn^2+^, and Fe^2+^. It is noted that MnO_2_ nanozyme was unable to determine ultralow metal ion concentration; thus, a more sensitive detecting assay should be developed in the follow-up study.

## Figures and Tables

**Table 1:** Analytical results for the detection of metal ion-contaminated water samples by MnO_2_ nanozyme-2,6-DMP detecting systems.

Real samples	Added Cu^2+^ (mmol·L^−1^)	Detected Cu^2+^ (mmol·L^−1^)	Recovery	RSD (*n* = 5)	Added Zn^2+^ (mmol·L^−1^)	Detected Zn^2+^ (mmol·L^−1^)	Recovery	RSD (*n* = 5)	Added Mn^2+^ (mmol·L^−1^)	Detected Mn^2+^ (mmol·L^−1^)	Recovery	RSD (*n* = 5)	Added Fe^2+^ (mmol·L^−1^)	Detected Fe^2+^ (mmol·L^−1^)	Recovery	RSD (*n* = 5)
Pond water 1	0.002	0.002	104.8%	6.9%	0.002	0.002	108.6%	7.3%	0.002	0.002	106.7%	5.4%	0.002	0.002	116.5%	13.2%
	0.01	0.010	101.1%	3.7%	0.01	0.013	111.2%	10.5%	0.01	0.009	98.6%	6.3%	0.01	0.011	114.9%	8.8%
	0.05	0.052	105.8%	5.4%	0.05	0.051	102.7%	3.8%	0.05	0.053	106.1%	7.4%	0.05	0.056	111.9%	26.1%
	0.25	0.253	106.6%	4.9%	0.25	0.0248	97.8%	4.3%	0.25	0.255	112.4%	11.2%	0.25	0.246	96.6%	12.0%

Pond water 2	0.002	0.002	106.9%	9.8%	0.002	0.002	95.0%	6.7%	0.002	0.002	106.5%	6.3%	0.002	0.002	110.5%	15.7%
	0.01	0.009	93.0%	11.2%	0.01	0.013	111.2%	5.3%	0.01	0.012	124.0%	11.8%	0.01	0.011	114.3%	8.6%
	0.05	0.054	108.6%	7.6%	0.05	0.052	104.4%	4.2%	0.05	0.047	94.5%	4.9%	0.05	0.048	97.2%	7.3%
	0.25	0.248	99.4%	3.3%	0.25	0.246	98.5%	3.1%	0.25	0.254	101.7%	2.2%	0.25	0.252	100.9%	3.5%

## Data Availability

The data used to support the findings of this study are available from the corresponding author upon request.
